# STGRNS: an interpretable transformer-based method for inferring gene regulatory networks from single-cell transcriptomic data

**DOI:** 10.1093/bioinformatics/btad165

**Published:** 2023-04-02

**Authors:** Jing Xu, Aidi Zhang, Fang Liu, Xiujun Zhang

**Affiliations:** Key Laboratory of Plant Germplasm Enhancement and Specialty Agriculture, Wuhan Botanical Garden, Chinese Academy of Sciences, Wuhan 430074, China; University of Chinese Academy of Sciences, Beijing 100049, China; Key Laboratory of Plant Germplasm Enhancement and Specialty Agriculture, Wuhan Botanical Garden, Chinese Academy of Sciences, Wuhan 430074, China; Key Laboratory of Plant Germplasm Enhancement and Specialty Agriculture, Wuhan Botanical Garden, Chinese Academy of Sciences, Wuhan 430074, China; Key Laboratory of Plant Germplasm Enhancement and Specialty Agriculture, Wuhan Botanical Garden, Chinese Academy of Sciences, Wuhan 430074, China; Center of Economic Botany, Core Botanical Gardens, Chinese Academy of Sciences, Wuhan, 430074 China

## Abstract

**Motivation:**

Single-cell RNA-sequencing (scRNA-seq) technologies provide an opportunity to infer cell-specific gene regulatory networks (GRNs), which is an important challenge in systems biology. Although numerous methods have been developed for inferring GRNs from scRNA-seq data, it is still a challenge to deal with cellular heterogeneity.

**Results:**

To address this challenge, we developed an interpretable transformer-based method namely STGRNS for inferring GRNs from scRNA-seq data. In this algorithm, gene expression motif technique was proposed to convert gene pairs into contiguous sub-vectors, which can be used as input for the transformer encoder. By avoiding missing phase-specific regulations in a network, gene expression motif can improve the accuracy of GRN inference for different types of scRNA-seq data. To assess the performance of STGRNS, we implemented the comparative experiments with some popular methods on extensive benchmark datasets including 21 static and 27 time-series scRNA-seq dataset. All the results show that STGRNS is superior to other comparative methods. In addition, STGRNS was also proved to be more interpretable than “black box” deep learning methods, which are well-known for the difficulty to explain the predictions clearly.

**Availability and implementation:**

The source code and data are available at https://github.com/zhanglab-wbgcas/STGRNS.

## 1 Introduction

Single-cell RNA-sequencing (scRNA-seq) technologies offer a chance to understand the regulatory mechanisms at single-cell resolution (Wen and Tang 2022). Subsequent to the technological breakthroughs in scRNA-seq, several analytical tools have been developed and applied towards the investigation of scRNA-seq data (Qi et al. 2020; Wang et al. 2020; Li et al. 2022b; Zhang et al. 2022). Most of these tools focus on the study of cellular heterogeneity (Chen et al. 2019; Luecken and Theis 2019). Gene regulatory networks (GRNs) constitute a crucial blueprint of regulatory mechanisms in cellular systems, thereby playing a pivotal role in biological research (Emmert-Streib et al. 2014; Lopes-Ramos et al. 2018; Jiang and Zhang 2022). Therefore, it is imperative to develop a precise tool for inferring GRNs from scRNA-seq data.

**Figure 1:**
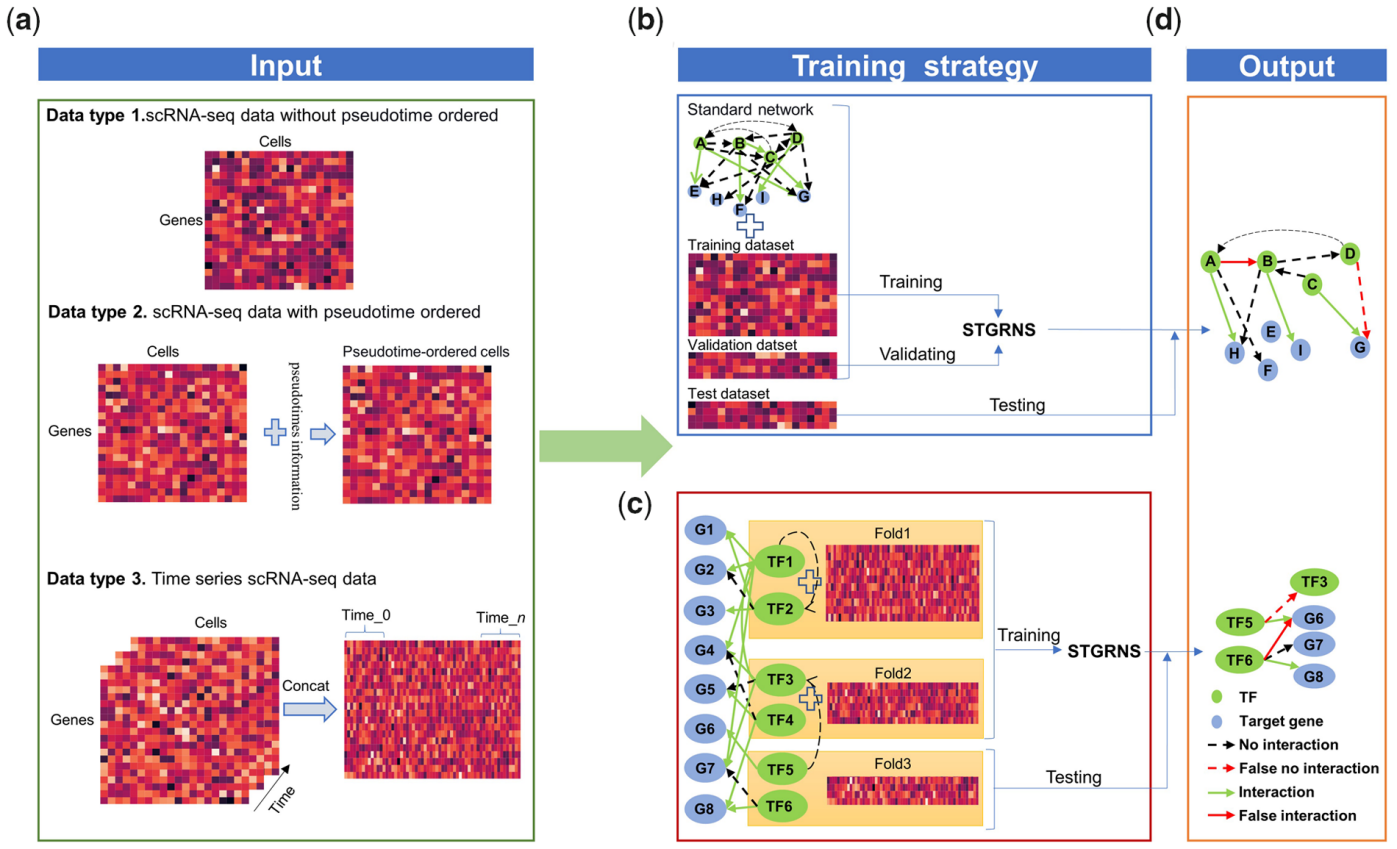
The workflow of STGRNS. (a) Three types of datasets that can be dealt with the STGRNS. Data type 1 is scRNA-Seq data without pseudo-time ordered cells. Data type 2 is scRNA-seq with pseudo-time ordered cells. Data type 3 is time-course scRNA-seq data. (b) The training strategy for the GRN reconstruction. The same TFs and genes exist in the training and testing datasets. The GRNs reconstruction adopts this strategy. (c) The training strategy for the TF–gene prediction. The training dataset and the test dataset have the same genes but not the same TFs. We demonstrate one loop using threefold cross-validation. The size of each fold is not equal because the size of the TGs of each TF is different. The TF–gene prediction adopts this strategy. (d) The output of STGRNS for network inference

The methods for inferring GRNs from scRNA-Seq data can be classified into unsupervised methods and supervised methods. Unsupervised methods use statistical and computational techniques to discover underlying patterns and structures within the scRNA-seq data, enabling the identification of regulatory interactions without known network (gene pairs) (Chan et al. 2017; Specht and Li 2017; Dai et al. 2019; Shu et al. 2021; Deshpande et al. 2022; Ferrari et al. 2022). While supervised methods depend on known network to train a model and then identify regulatory interactions between genes (Yuan and Bar-Joseph 2019, 2020; Chen et al. 2021; Chen and Liu 2022; Li et al. 2022; Xu et al. 2022; Zhao et al. 2022; Zhang et al. 2023). For unsupervised methods, such as DeepSEM (Shu et al. 2021), PIDC (Chan et al. 2017), SINGE (Deshpande et al. 2022), and LEAP (Specht and Li 2017), the disadvantages of high sparsity, noise, and dropout events in scRNA-Seq data limit their ability of inferring GRNs (Pratapa et al. 2020; Nguyen et al. 2021). What’s worse, most unsupervised methods are nonuniversal (Marbach et al. 2012), i.e. they only perform well on one of three type of datasets including scRNA-seq data without pseudo-time ordered cells, scRNA-seq data with pseudo-time ordered cells and time-series scRNA-seq data (Fig. 1a) (Marbach et al. 2012). Supervised methods, such as convolutional neural network for coexpression (CNNC) (Yuan and Bar-Joseph 2019), DGRNS (Zhao et al. 2022), and TDL (Yuan and Bar-Joseph 2021), have been devised to address the expanding scale and intrinsic complexity of scRNA-seq data, with deep learning models being commonly employed (Erfanian et al. 2021). In general, the workflow for the inference of GRNs from scRNA-Seq data based on deep learning approaches comprises two primary steps, i.e. the conversion of gene pairs to image data and the classification of the resultant image data into interaction or no-interaction categories by employing convolutional neural network (CNN) models.

There exist certain limitations to the employment of CNN model-based approaches for GRN reconstruction. First of all, the generation of image data not only gives rise to unanticipated noise but also conceals certain original data features. Secondly, this procedure is time-consuming, and since it alters the format of scRNA-seq data, the outcomes predicted by these computational approaches utilizing CNNs cannot be wholly elucidated. Nevertheless, CNN-based models have achieved notable success in various biological tasks (Deng et al. 2021; Greener et al. 2022). In general, CNN-like models lack of ability to capture global information due to their limited receptive (Khan et al. 2020). Additionally, it takes a very long time to train CNN-like models, especially for large datasets. Some methods have been proposed to combine CNN-like and recurrent neural network (RNN)-like models for inferring GRNs, but they are time-consuming and inferior to CNN-based models in certain cases (Yuan and Bar-Joseph 2021; Zhao et al. 2022). Recently, some methods based on graph neural networks were developed to infer GRNs from scRNA-seq data (Chen and Liu 2022; Li et al. 2022).

There are two different training strategies for supervised methods to infer GRNs. The first strategy is to divide benchmark datasets into training datasets, validation datasets, and test datasets based on dataset size, followed by leave-one-out cross-validation (Fig. 1b) (Zhao et al. 2022). The second strategy is to divide them into 3-fold based on the number of transcription factors (TFs) in benchmark datasets, and adopt 3-fold cross-validation (Yuan and Bar-Joseph 2019; Chen et al. 2021; Yuan and Bar-Joseph 2021; Xu et al. 2022). It means that all samples related to a particular TF are used either in training or solely in testing datasets (Zhang et al. 2013). Henceforth, we shall denote all samples related to a particular TF together with their corresponding target genes (TGs) as (TF, TGs). Based the information of regulators and targets, GRNs can be taken as two types of networks, i.e. gene–gene network and TF–gene network. Generally, the gene–gene network reconstruction task is matched with the first strategy in Fig. 1b and the TF–gene network prediction task is matched with the second strategy in Fig. 1c.

To address the above issues, we developed a supervised method, namely STGRNS, based on transformer (Vaswani et al. 2017). In this algorithm, gene expression motif (GEM) was proposed to convert each gene pair into the form that can be fed as input to the transformer encoder architecture (Supplementary Note S1). STGRNS can accurately infer GRNs based on known relationships between genes, irrespective of whether the data are static, pseudo-time, or time-series. To evaluate the performance of STGRNS, we compare it with other state-of-the-art tools on 48 benchmark datasets, including 21 static scRNA-seq dataset (18 unbalanced datasets for the gene–gene network reconstruction and 3 balanced datasets for the TF–gene network prediction) and 27 time-series scRNA-seq dataset (23 balanced datasets for the gene–gene network reconstruction and 4 balanced datasets for the TF–gene network prediction). All the results show that STGRNS is superior to other methods as a deep learning-based method. What is more, unlike other “black box” deep learning-based methods, which are often characterized by their opacity and associated difficulty in furnishing lucid justifications for their predictions, STGRNS is more reliable and can interpret the predictions.

## 2 Materials and methods

### 2.1 The structure of STGRNS

**Figure 2:**
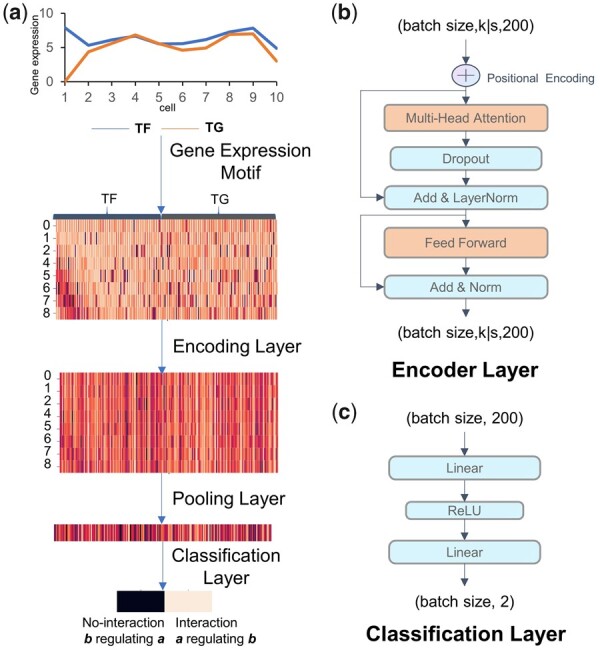
The overall architecture of STGRNS. (a) The processing flow of each gene pair. (b) The encoder layer and (c) the classification layer in STGRNS

The structure of STGRNS includes four distinct modules, i.e. the GEM module, the positional encoding layer, the transformer encoder, and the classification layer. The GEM module is responsible for reconfiguring gene pairs into a format that can be utilized as input by the transformer encoder. The positional encoding layer is utilized to capture the positional or temporal information (Fig. 2a). The transformer encoder is used for calculating the correlation of different sub-vectors. It pays more attention to key sub-vectors (Fig. 2b). The classification layer is used to produce the final classification output (Fig. 2c).

#### 2.1.1 Gene expression motif

**Figure 3:**
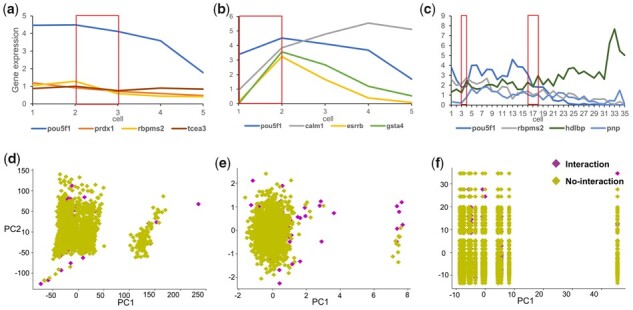
Effectiveness of GEM. Average gene expression of each 100 cells. (a) The scRNA-seq data without timing information. (b) The scRNA-seq data with pseudo-timing information. (c) The scRNA-seq data with timing information. In all three cases, pou5f1 was selected as the TF. (d–f) The plot of the 2D PCA. The 500_Nonspecific-ChIP-seq-network_ mESC-GM dataset was processed by three different input generation methods. The PCA function is provided by scikit-learn (Pedregosa et al. 2011) and we use its default parameter values. (d) The 2D plot of scRNA-seq data processed by the input generation method of CNNC. (e) The 2D plot of scRNA-seq data processed by the input generation method of DGRNS. (f) The 2D plot of scRNA-seq data processed by GEM

Notably, the phenomenon that expression values of genes that are regulated by the shared TF are synchronous for some spans/phases is easily observed (Fig. 3a–c). With this assumption, we propose a new data processing technique namely GEM to reintegrate the gene pairs into the form that can be used as input of the transformer encoder layer. Let *X_i_* = (*X_i_*_,__1,_*X_i_*_,__2,_ … … _,_*X_i_*_,_*_k_*) represents the expression vector of gene *i*, including *k* cells. *X_i_* is divided into contiguous sub-vectors of length *s*, where the selection of the sub-vector length is determines by the “window size” parameter. The *l*-th sub-vector of gene *i* is denoted by *X_i_*_,_*_l_*. Formally, *X_i_*_,_*_l_*=(*X_i_*_,_*_l_*_×s+0,_*X_i_*_,_*_l_*_×__*s*__+1, … … ,_*X_i_*_,_*_l×s+s−1_*), *l*∈[1 … … ,*k*|*s*]. After the above operation, gene *i* (*X_i_*) and gene *j* (*X_j_*) within a gene pair (*X_i_*_,_*X_j_*) can be described as vectors, denoted by *X_i_*=(*X_i_*_,0,_*X_i_*_,1, … … ,_*X_i_*_,_*_k|s_*) and *X_j_*=(*X_j_*_,0,_*X_j_*_,1, … … ,_*X_j_*_,_*_k_*_|__*s*_), respectively. For each sub-vectors in *X_i_* and *X_j_*, we concat them into one sub-vector, denoted by *X_ij, m,_* m∈[0 … … , *k*|*s*]. Formally, *X_i_* and *X_j_* are concated to form a new vector *X_ij_*=(*X_ij_*_,__0,_*X_ij_*_,__1, … … ,_*X_ij_*_,_*_k|s_*), which is used as the input of the transformer encoder.

The conversion of gene pairs into the input format of the transformer encoder by GEM presents a novel method for constructing GRNs based on scRNA-seq data using deep learning model. This approach represents an innovative pattern that is expected to have significant implications in the field of scRNA-seq analysis.

#### 2.1.2 Function for positional encoding

We put *X_ij_* into the transformer encoder at one time, which makes the order information or time information of gene expression vectors lost. We use the sine function to represent the odd-numbered sub-vectors and the cosine function to represent the even-numbered sub-vectors as demonstrated follows where *m* represents the position of *X_ij, m_* in *X_ij_*, 2*n* represents the even sub-vectors, and 2*n*+1 represents the odd sub-vectors.


(1) $$\text{PE}(m,2n)=\text{sin}(m/10\;000^{2n/s}),$$

(2) $$\text{PE}(m,2n+1)=\text{cos}(m/10\;000^{(2n+1)/s}),$$

Additionally, an ablation experiment was conducted to investigate the impact of positional encoding on the performance of STGRNS. The results indicated that STGRNS had reduced performance when positional encoding was omitted, as shown in Supplementary Fig. S10. Nevertheless, even without positional encoding, STGRNS outperformed other methods. The input of encoder layer *X*_posi_ is defined as *X_ij_* plus the *X_ij_* processed by positional encoding, as demonstrated below,


(3) $$X_{\text{posi}}=X_{ij}+\text{Positional}(X_{ij}).$$

#### 2.1.3 Function for encoder layer

In this work, we only use one transformer encoder layer consisting of two sub-networks. One sub-network is a multi-head attention network and another one is a feed-forward network. Several special properties of the attention mechanism contribute greatly to its outstanding performance. One of them is to pay much attention to vital sub-vectors of gene expression vectors, which is in line with the GEM we proposed. Benefiting from this mechanism, STGRNS can ignore the adverse effects caused by insignificant sub-vectors. Another advantage is that it can capture connections globally, which means that it can make full use of discontinuous sub-vectors to improve the accuracy of STGRNS. The attention mechanism employed in STGRNS is the Scaled Dot-Product Attention. This is done by computing a weighted sum of the sub-vectors, where the weights are determined by a softmax function, applied to a compatibility function that measures the similarity between the current sub-vector and the other sub-vectors in the gene pairs, where *Q* *=* *W^q^X*_posi_, *K* *=* *W^k^X*_posi_, *V* *=* *W^v^X*_posi_, the *W^q,k,v^* is the linear project weight, $\text{softmax}(z_{i})=\text{exp}(z_{i}/\sum_{j}z_{j})$.


(4) $$\text{Attention}(Q,K,V)=\text{softmax}(Q\cdot K^{T}/\sqrt{S}),$$

Although RNNs-based model can also directly handle sub-vectors, they lack the capability to capture long-term dependencies. In addition, STGRNS can parallelize the calculation, which is one of the main reasons why STGRNS runs fast in most datasets. Moreover, compared with CNN- and RNN-based models, it is more efficient and has fewer parameters under the same condition (Vaswani et al. 2017). We use multi-head self-attention to capture different interaction information in multiple different projection spaces. We set the hyperparameter head to 2 in STGRNS, which means we have two parallel self-attention operations where


(5) $$\text{MulitHead}(Q,K,V)={\text{Concat}(\text{head}}_{1}{,\text{head}}_{2}),$$

(6) $${\text{head}}_{h}=\text{Attention}(Q_{h},K_{h},V_{h}).$$

The *X*_posi_ after multi-head attention and processed by residual connection and layer normalization is converted into *X*_attention_ as the input of the feed-forward network.


(7) $$X_{\text{attention}}=\text{LayerNorm}(X_{\text{posi}}+X_{\text{attention}})$$

Although self-attention can use adaptive weights and focus on all sub-vectors, there are still some nonlinear features not captured. Therefore, the feed-forward network is to increase nonlinearity. The feed-forward layer contains two linear layers with the rectified linear activation function (ReLU) as the activation function


(8) $$X_{\text{encoder}}=\max(0,X_{\text{attention}}W_{1}+b_{1})W_{2}+b_{2}.$$

#### 2.1.4 Function for classification layer

The average pooling layer utilized by STGRNS is expressed by


(9) $$X_{\text{average}}=\text{mean}(X_{\text{encoder}}).$$

For the classification layer, we used two linearly connected networks and ReLU as the activation function. The classification of the input data is performed by


(10) $$X_{\text{predict}}=\max(0,X_{\text{average}}W_{1}+b_{1})W_{2}+b_{2}.$$

Despite the simplicity of the classification layer, it can yield flawless outcomes through the GEM, even in the absence of the transformer encoder layer (Supplementary Fig. S12). We used the sigmoid function

$S(X_{\text{predict}})=1/\left(1+e^{-x_{\text{predict}}}\right)$ for binary classification and the Adaptive Momentum Estimation algorithm as the optimizer.

### 2.2 Benchmark datasets

In this study, two kinds of datasets including small-scale unbalanced datasets and large-scale balanced datasets are used for analysis. The unbalanced datasets include seven scRNA-seq datasets derived from the BEELINE framework (Pratapa et al. 2020). The genes that are expressed in fewer than 10% of cells are filtered out. We only select the top 500 and 1000 highly variable genes from these scRNA-seq datasets. In addition, we configure three standard networks for each dataset, which are cell-type-specific ChIP-seq (The ENCODE Project Consortium 2012; Xu et al. 2013; Davis et al. 2018; Oki et al. 2018), nonspecific ChIP-seq (Han et al. 2015; Liu et al. 2015; Garcia-Alonso et al. 2019), and functional interaction networks collected from the STRING database (Szklarczyk et al. 2019). About 41 unbalanced benchmark datasets including 18 static and 23 time-series scRNA-seq dataset were used for this study (Supplementary Table S1).

The balanced datasets include mouse embryonic stem cells (mESCs), bone marrow-derived macrophages(bone), dendritic cells(dendritic), hESC(1), hESC(2), mESC(1), and mESC(2) (Yuan and Bar-Joseph 2019, 2021). Genes that do not express in all cells are filtered out. The standard networks are derived from cell-type-specific ChIP-seq of CNNC and TDL, respectively. For the causality prediction task, (***a***, ***b***) where ***a***regulate ***b*** will be assigned 1 while the label for (***b***, ***a***) is 0. For the interaction prediction task, ***a* (*b*)** and its regulated ***b* (*a*)**, (***a***, ***b***) will be assigned a label of 1 while the label for (***a***, ***c***) is 0 where **c** is a gene randomly selected from the genes not regulated by ***a***. In the end, we prepared seven balanced benchmark datasets including three static and four time-series scRNA-seq dataset (Supplementary Table S2).

### 2.3 Training and testing strategy

For the gene–gene network reconstruction task, we divide benchmark datasets into training, validation, and test datasets with 3:1:1 ratio and maintain the same positive-to-negative ratio on each dataset. The training dataset is used to train the model, the validation dataset is used to select hyperparameters, and the model is evaluated on the test dataset. The supervised algorithm for comparison is also based on this strategy. We evaluate the performance of the unsupervised algorithm on test datasets.

For the TF–gene prediction task, we adopted 3-fold cross-validation on the TF–gene network prediction task. We divided datasets into the training and test datasets according to the number of TFs and divided 20% of the training datasets as validation datasets. This strategy is followed by all the methods involved in the comparison.

### 2.4 Metrics for evaluation

To evaluate the performance of the proposed method, the following measures are used. True positive rate (TPR) and false positive rate (FPR) are used to plot the receiver operating characteristic (ROC) curves, and the area under the ROC curve (AUROC) is calculated. Precision and Recall are also used to draw the PR curve, and the area under the precision–recall curve (AUPRC) refers to the area under the PR curve (Zhang et al. 2012, 2015). Mathematically, they are defined as FPR=FP/(FP+TN), TPR=TP/(TP+FN), and Recall=TP/(TP+FN), where TP, FP, TN, and FN are the numbers of true positives, false positives, true negatives, and false negatives, respectively.

## 3 Results

### 3.1 Effectiveness of GEM

For most deep learning-based methods, gene pairs are usually transformed into the form matching with the training model. This process is generally called input generation. A simple but effective input generation method not only considerably preserves the features of the scRNA-seq data, but also achieves perfect results on different types of datasets. For example, CNNC method only achieves competitive results on a few datasets using its input generation method (Supplementary Note S2). The performance of CNNC is greatly improved using another input generation method but is still weaker than our approach (Supplementary Fig. S1). The input generation method in DGRNS consumes much time (Supplementary Note S3). Therefore, it is not suitable for large-scale data. What is worse, there are too many parameters for input generation method in DGRNS and these parameters have to be adjusted according to the number of cells.

To compare the effectiveness of input generation methods, we selected the input generation methods of CNNC and DGRNS to pre-process the top 500 highly variable genes mHSC-GM scRNA-seq dataset with nonspecific cell networks. Figure 3d–f shows the plot of 2D principal components analysis (PCA) of the scRNA-seq data processed by these input generation methods. The number of interaction samples is much smaller than the number of no-interaction samples, making it more difficult to distinguish between them. Besides, it is evident that the dataset is linearly inseparable, so linear model-based methods are unsatisfactory. It is similar for the three input generation methods to distinguish between interaction and no-interaction samples. However, we find that the scRNA-seq data processed by GEM is subdivided clearly into seven columns (Fig. 3f). Each column represents samples with the same TFs, illustrating that the differences between TFs are not masked by GEM. In contrast, other input generation methods conceal this feature. Moreover, GEM takes less time and costs less storage when the number of cells is not very large (Supplementary Fig. S2a–f). The time and storage consumed by GEM increase with the number of cells and genes augmenting, while the time and storage consumed by the input generation method of CNNC increase with the number of genes augmenting (Supplementary Fig. S2g).

### 3.2 Effectiveness of STGRNS in gene–gene network inference

To evaluate the effectiveness of STGRNS, the experiment was firstly implemented on the task of inferring gene–gene regulatory networks from scRNA-seq data. We compared STGRNS with unsupervised methods and supervised methods on the unbalanced datasets. The unsupervised methods include PIDC, DeepSEM, LEAP, and SINGE, and the supervised methods include CNNC and DGRNS. PIDC is an unsupervised method to infer GRNs using partial information decomposition between genes. Based on beta-variational autoencoder, DeepSEM can deal with various single-cell tasks including GRN inference, scRNA-seq data visualization, cell-type identification, and cell simulations. LEAP is an unsupervised method based on Pearson’s correlation. As an unsupervised method, SINGE applies kernel-based Granger causality regression to alleviate irregularities in pseudo-time scRNA-seq data. The central architecture of CNNC is VGGnet (Simonyan and Zisserman, 2014) (Supplementary Note S4). DGRNS is a hybrid method combining CNN and RNN (Supplementary Note S5). We divided benchmark datasets into training datasets, validation datasets, and test datasets with the ratio of 3:1:1. We assessed the performances of the unsupervised algorithms on the test datasets. The AUROC and AUPRC were used as evaluation scores.

**Figure 4:**
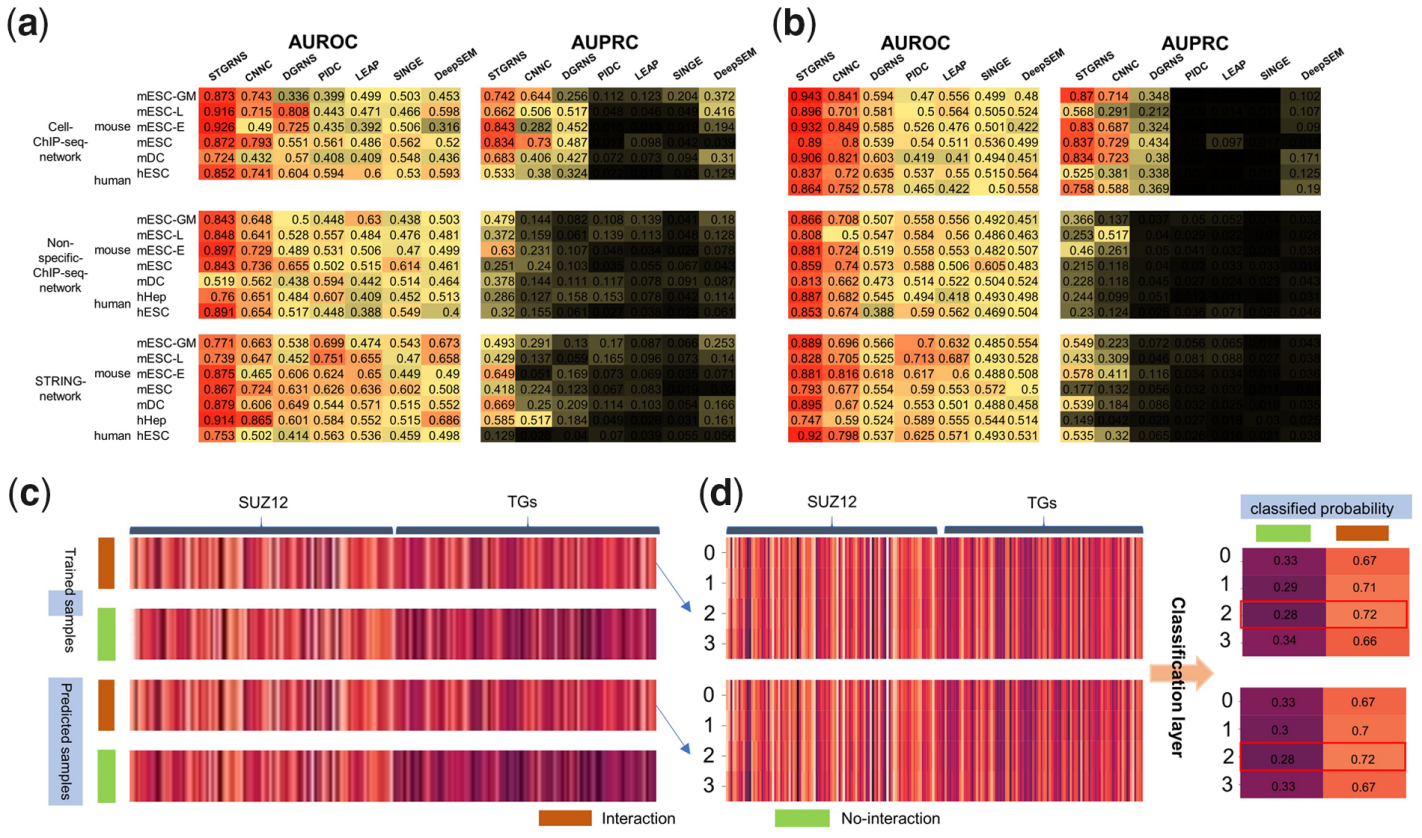
Comparison of STGRNS with other methods and the interpretation of STGRNS on the GRN reconstruction task. (a) Comparison results on the top 500 highly variable genes datasets. (b) Comparison results on the top 1000 highly variable genes datasets. (c) Heatmap of true positive prediction samples after pooling layer in training datasets and test datasets. (d) Heatmap of the trained interaction samples and predicted interaction samples after the encoder layer and let each sub-vector pass through the classification layer to get the probability that each sub-vector is classified as 1

As shown in Fig. 4a and b, the unsupervised methods are worse than the supervised methods among which STGRNS performs the best. When using the AUROC ratio metric, STGRNS achieves significant performance on 95.12% (39/41) of benchmark datasets. Besides, it is 14.42% higher than the second-best AUROC value and is around 90% on most datasets. In the term of AUPRC, STGRNS outperforms all other algorithms on every dataset and is 18.88% higher on average than the second-best AUPRC value. Remarkably, no method is better than STGRNS on the top 1000 highly variable genes datasets. We also draw box plots of the AUC of all algorithms on the top 500 and 1000 datasets to investigate how the performance of these methods is influenced by datasets of varying sizes and numbers of cells. Overall, the unsupervised methods are more robust than the supervised methods on most datasets. For supervised methods, the performance of STGRNS is more stable than CNNC and DGRNS in the term of AUROC, but the result is reversed in the term of AUPRC. Furthermore, STGRNS runs faster than other supervised models on 53.66% (22/41) of benchmark datasets (Supplementary Fig. S3).

We also make the comparison on the same datasets without pseudo-time ordered cells. As a result, the pseudo-times have a little impact on the performance of STGRNS. STGRNS performs better than other methods on 95.12% (39/41) of the benchmarks in the term of AUROC and on 94% (38/41) of the benchmarks in the term of AUPRC, indicating that it can adapt to different types of datasets and achieve the best performance (Supplementary Fig. S4a and b). In order to assess the resilience of STGRNS towards varying positive-to-negative ratios, an exhaustive evaluation of its performance was conducted on a representative dataset. The dataset consisted of the top 1000 highly variable genes from hHep scRNA-seq dataset, with a nonspecific-ChIP-seq-network utilized as a template. Positive-to-negative ratios were varied across 10 levels (10:1, 8:1, 5:1, 3:1, 2:1, 1:2, 1:3, 1:5, 1:8, and 1:10). Overall, although the performance of STGRNS was marginally subpar, it still achieved commendable results (Supplementary Fig. S4c). Furthermore, in order to investigate the impact of single-cell coverage on the performance of STGRNS, we randomly sampled transcripts from 10% and 90% of transcript reads in the hHep scRNA-seq dataset using Scanpy (Wolf et al. 2018) while maintaining the same benchmark network. Results indicate that STGRNS can effectively infer the GRN even at extremely low down-sampling rates. Compared to the other two SOAT methods (DGRNS and CNNC), STGRNS exhibits a greater degree of stability in the face of scRNA-seq coverage variability (Supplementary Fig. S4d). All of the outcomes suggest that the STGRNS approach is capable of precisely inferring GRNs.

### 3.3 The interpretation of STGRNS in gene–gene network inference

The supervised methods learn features from labelled data and then use the learned features to judge unlabelled data. We can find that the GRN inference approaches based on supervised models, no matter what kind of scRNA-seq data, can infer accurate GRNs as long as there is enough labelled data. To explain what STGRNS has learned, we used the top 1000 highly variable genes in mESC scRNA-seq dataset with the cell-ChiP-seq-network as a template. We randomly select one interaction sample and one no-interaction sample, respectively, from the training datasets and test datasets of (suz12, TGs). We can see that the expression level of TFs and TGs are similar in interaction samples, but they are different in no-interaction samples (Fig. 4c). This is the main difference between interaction and no-interaction samples. To further explore which sub-vectors of the gene expression value vectors in (suz12, TGs) play a crucial role in the GRN reconstruction task, we add the classification layer after the encoder layer and visualize them after the classification layer (Supplementary Fig. S10c right). Figure 4d reveals that the third sub-vector has the most vital impact on the final result in the trained and predicted samples. To further prove the reliability of this conclusion, we calculate the number of the most important sub-vector in all samples of suz12 in the training and test dataset. After analysis, the number that the third sub-vector is the most vital accounts for 69.02% in the training dataset and 77.66% in the test dataset, which fits our conclusion and also confirms the effectiveness of GEM. In addition, to prove that the third sub-vector is not the most important sub-vector of all TFs, we analysed all samples of pou5f1, of which the number that the fourth sub-vector is the most important accounts for 85.64% in the training dataset and accounts for 85.71% in the test dataset. These confirm that the operation of GEM can improve the accuracy of the model and can greatly benefit the interpretation of the predicted results of STGRNS.

### 3.4 Effectiveness of STGRNS in TF–gene network prediction

To further prove the extensibility and generality of STGRNS, we validate our model on the TF–gene prediction task. It contains the interaction prediction task and the causality prediction task. For a gene pair (***a, b***), determining whether there is a relationship between ***a*** and ***b*** is the interaction prediction and judging the direction of regulation between ***a*** and ***b*** is the causality prediction. We set the label of interaction and ***a*** regulating ***b*** as 1 and the opposite as 0. Following previous studies (Yuan and Bar-Joseph 2019, 2021), we conducted 3-fold cross-validation to evaluate the performance of STGRNS on the balanced datasets. The results proved that supervised methods performed better than unsupervised methods on these datasets, so we choose supervised approaches to make the comparison including CNNC and TDL. TDL is a computational method that extends the CNNC and is intended for the analysis of time-course scRNA-seq data, including 3D Convolutional Neural Network (3D_CNN) and LSTM (Supplementary Note S6). TDL converts the time-course scRNA-seq data into a 3D tensor representation that serves as the input of the method. The 3D_CNN architecture comprises a tensor input layer with dimensions T × 8 × 8, followed by multiple 3D convolutional layers, max pooling layers, a flatten layer, and a classification layer with unspecified dimensions.

**Figure 5:**
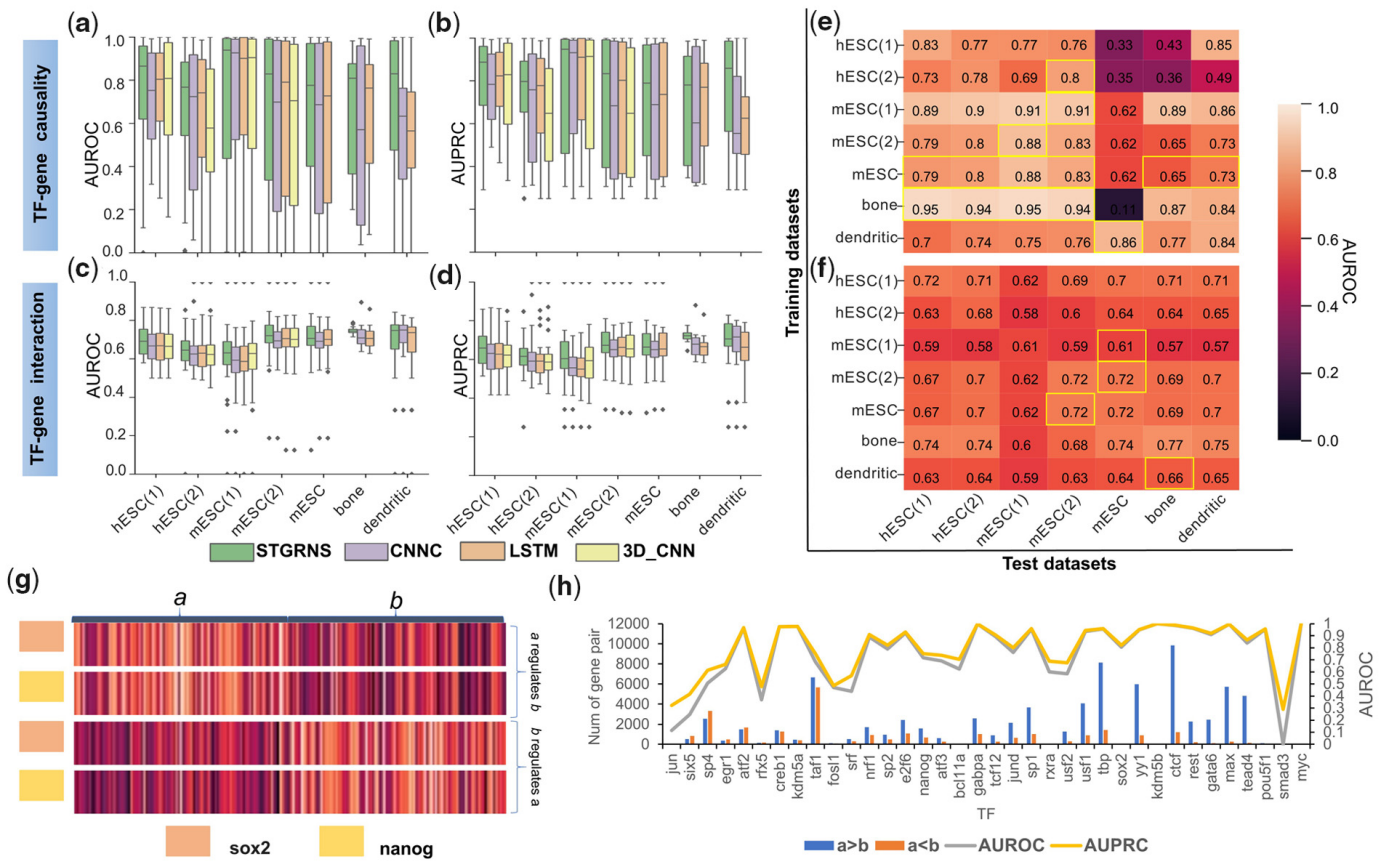
Comparison of STGRNS with other methods and the interpretation of STGRNS on the TF–gene prediction task. (a and b) Comparison results on the TF–gene causality task. (c and d) Comparison results on the TF–gene interaction task. (e) Transferability of STGRNS on the causality prediction task. (f) Transferability of STGRNS on the interaction prediction task. (g) Two sets of samples. (h) The performance of each TF and the number of ***a* **>** *b***, ***a* **<** *b*** in gene pairs

As a result, STGRNS performed the best among these computational methods on the TF–gene prediction task across the overwhelming majority of datasets. For the causality prediction task, STGRNS achieves the best on all seven benchmark datasets in terms of both AUROC and AUPRC (Fig. 5a and b). For the interaction prediction task, STGRNS achieves the best performance on 85.71% (6/7) of benchmark datasets in terms of both AUROC and AUPRC ratio metrics (Fig. 5c and d). STGRNS also costs less training time than other methods on 57.14(4/7) of benchmark datasets on the causality prediction task (Supplementary Fig. S3c). Unlike the gene–gene network reconstruction task, STGRNS learns the general features from samples in the training datasets to distinguish between interaction, no-interaction, and ***a*** regulating ***b***, ***b*** regulating ***a***. For the TF–gene network prediction task, the performance of STGRNS increases by an average of 25.64% on the causality prediction task and increases by an average of 3.31% on the association prediction task in the term of AUROC (Supplementary Fig. S5). Then, we trained STGRNS using one dataset and then test the performance of STGRNS on other datasets. As a result, STGRNS has a certain transferability (Fig. 5e and f). We assume that if there is enough pre-training data, it can accurately predict TF–gene interactions and causality for other species with fewer labelled data.

### 3.5 The interpretation of STGRNS in TF–gene network prediction

To understand the major feature, which STGRNS learns to distinguish the regulation direction, we chose the hESC(1) as an example dataset. We randomly select the true positive predictions samples from (sox2, TGs) and (nanog, TGs), and then visualize them after the pooling layer (Fig. 5g). It is clear that the expression level of the TG ***b*** is significantly smaller than that of TF ***a***. We also count the size of true positive predictions in the hESC(1). Further, we calculate the number of gene expression values of ***a*** greater than ***b*** in the true positive predictions set and show the performance of each (TF, TGs) (Fig. 5h). In general, the size of gene pairs whose gene expression level of ***a*** is greater than ***b*** is proportional to the values of AUROC and AUPRC. Other scRNA-seq datasets also have the same phenomenon (Supplementary Fig. S6). We also use STGRNS trained on causality datasets to judge the interaction prediction task. However, the performance is very poor (Supplementary Fig. S7), which proves that the main feature which STGRNS learned from causality datasets is to distinguish regulation direction. In conclusion, we can confirm the main point STGRNS learned to judge the direction of regulation is that genes with smaller expression values are regulated.

### 3.6 Robustness of STGRNS to hyperparameters

**Figure 6:**
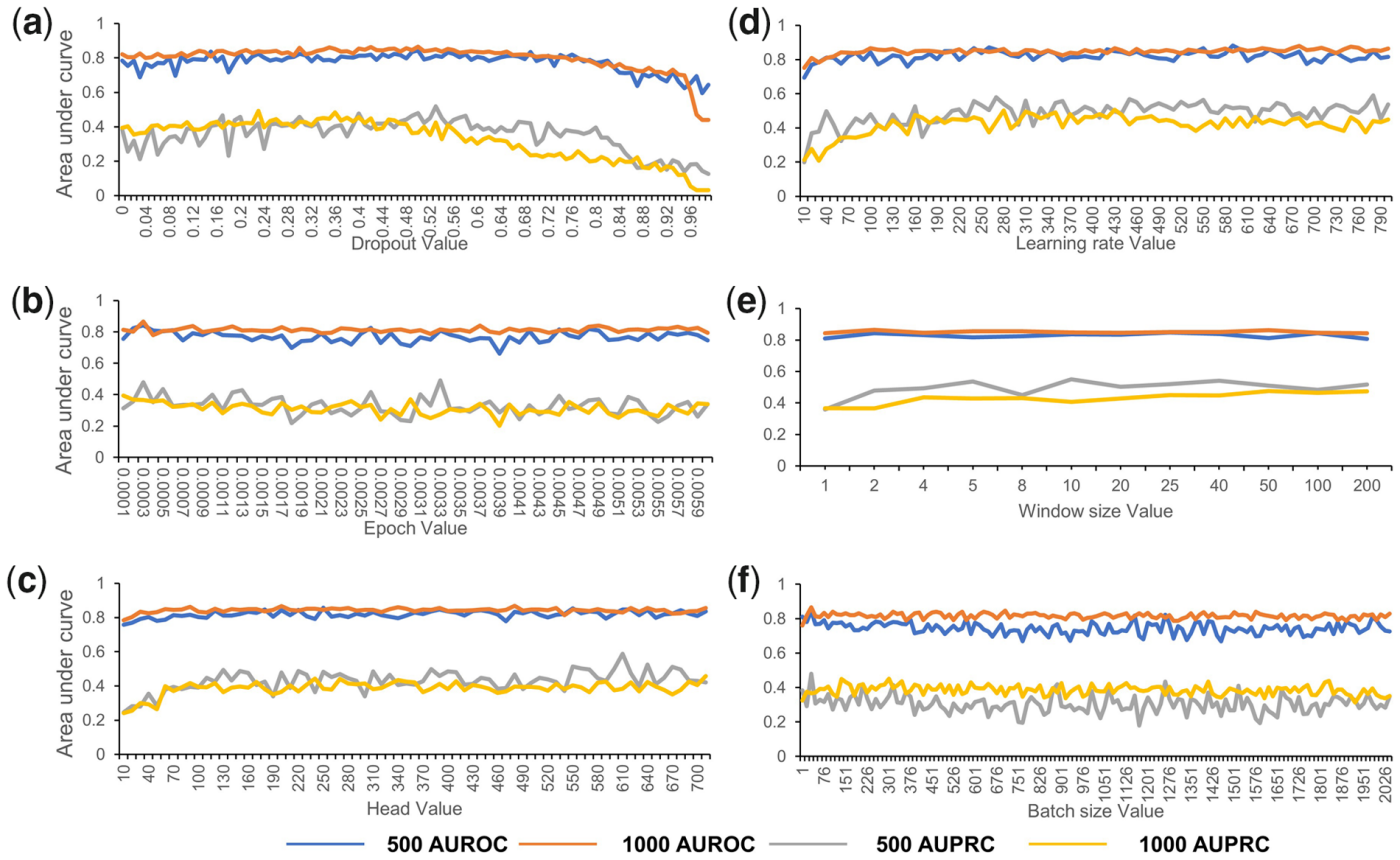
Parameter sensitivity analysis. STGRNS is robust to various parameters including dropout, learning rate, epoch, window size, head, and batch size. Performance is measured as AUROC and AUPRC. (a) The parameter for dropout ranges from 0.00 to 0.99 and each increment is 0.01. (b) The parameter for learning rate ranges from 0.0001 to 0.0059 and each increment is 0.0001. (c) The parameter for epoch ranges from 10 to 700 and each increment is 10. (d) The parameter for window size ranges from 10 to 790 (the number of cells in this dataset is 790) and each increment is 10. (e) The parameter for the head ranges from 1 to 200. (f) The parameter for batch size ranges from 1 to 2041 and each increment is 15

STGRNS has fewer hyperparameters compared to other GRN reconstruction methods based on deep learning models, which is one of the main reasons for its outstanding generalization. Most approaches tend to perform well on benchmark datasets, but their performance is often not satisfactory when they are applied to other datasets. One reason is that models are not generalized enough. Another reason is that models are not stable enough. In the other work, the changes in hyperparameters that are set before the learning process will greatly affect outcomes of models. Therefore, to assess the robustness of STGRNS to hyperparameters, we conduct sensitivity analysis for hyperparameters including dropout, learning rate, epoch, window size, head, and batch size in the STGRNS (Supplementary Table S3). We selected the mHSC-GM dataset with nonspecific-ChIP-seq-network and hESC(2) as templates. We modify one of the hyperparameters each time and set other hyperparameters as default values (Supplementary Table S3). Overall, changes in these hyperparameters have minimal effect on the performance of STGRNS, demonstrating that SGRNS is robust to hyperparameters (Fig. 6).

## 4 Discussion

With the rapid development of high-throughput technologies over the past two decades, numerous efficient computation methods for inferring GRNs have been proposed under the aegis of Dialogue for Reverse Engineering Assessments and Methods project (Marbach et al. 2012). Notably, these approaches have demonstrated remarkable efficiency in the age of bulk RNA-seq (Huynh-Thu et al. 2010; Zhang et al. 2012; Moerman et al. 2019). Furthermore, the accumulation of many known GRNs has resulted from the aforementioned efforts (Xu et al. 2013; Han et al. 2015; Liu et al. 2015; Oki et al. 2018; Szklarczyk et al. 2019; Fang et al. 2021). Given the rapid advancements in single-cell sequencing, it has become possible to investigate biological questions at the single-cell level (Ren et al. 2021). However, due to the sparse, high dimensionality, and high level of dropouts event inherent in scRNA-seq data, traditional methods for inferring GRNs face significant challenges in effectively inferring accurate GRNs. Although several methods based on supervised deep learning models have alleviated these problems, there is still much room to be improved.

In this work, we developed a performant and comprehensive framework called STGRNS for inference of GRNs from scRNA-seq data. We objectively and comprehensively validated our model on 48 benchmark datasets involving different species, lineages, network types, and network sizes. STGRNS consistently outperformed three widely used supervised models (CNNC, DGRNS, and TDL), as well as four unsupervised methods (PIDC, LEAP, SINGE, and DeepSEM) on the tasks of GRNs reconstruction and TF–gene prediction. STGRNS can also achieve superior performance compared to TDL methods that are specifically tailored for time-series data, across four distinct time-series datasets. In addition, STGRNS has certain transferability on the TF–gene prediction task (Supplementary Fig. S9). Specifically, STGRNS can partially address the challenges associated with limited information on known gene pairs. Besides, the analysis of trained and predicted samples can provide insight into the rules learned by the STGRNS from known gene pairs, which can enhance the reliability and trustworthiness of STGRNS. What is more, STGRNS exhibits a greater degree of hyperparameter robustness, which ensures that the algorithm can achieve satisfactory performance on a wider range of datasets.

The exceptional performance of STGRNS in the aforementioned tasks can be attributed to its technical advantages. Firstly, STGRNS stands out from other deep learning-based methods for GRN reconstruction by leveraging the Transformer, which has achieved state-of-the-art results in many artificial intelligence tasks (Liu et al. 2021) and has been widely adopted in the analysis of DNA sequences, protein sequences, and scRNA-seq data (Zeng et al. 2018; Clauwaert et al. 2021; Jumper et al. 2021; Chu et al. 2022; Shu et al. 2022; Yang et al. 2022; Chen et al. 2023), owing to its powerful attention mechanism. The attention mechanism employed by STGRNS is based on a self-attention mechanism, which enables the STGRNS to focus on distinct sub-vectors within the gene pair and compute a representation for each sub-vector. Secondly, STGRNS employs GEM to convert a gene pair into contiguous sub-vectors, which is a more time-consuming yet effective method compared to other methods used in CNNC and DGRNS. Remarkably, a noteworthy phenomenon is the synchronous expression pattern exhibited by various TGs under the regulation of a shared TF for certain intervals/phases. To capture and weight these synchronized phases, the STGRNS is trained by known gene pairs. This enables STGRNS to remember the synchronized phases. If a gene shows a similar trend with the TF in the learned synchronized phases, the STGRNS determines it to be a highly probable TG for the TF. It is the integration of GEM and attention mechanisms that allow STGRNS to infer accurately GRNs from scRNA-Seq data.

Despite these successful results, there are several aspects in which STGRNS can be improved. It is a general problem for supervised algorithms that they cannot achieve perfect performance when labelled data are insufficient. Meta learning (Zhang et al. 2023), transfer learning (Weiss 2016; Liu and Zhang 2022), and few-shot learning (Fang et al. 2021) are often used to solve the problem of few labelled data, so they are expected to overcome this problem in GRN reconstruction. The current research field is expanding rapidly, and we plan to enhance and extend STGRNS by incorporating diverse sources, such as scATAC-seq (Li et al. 2022a) and spatial transcriptomics (Yuan and Bar-Joseph 2020; Li et al. 2022b; Zheng et al. 2022), which will enable STGRNS to accurately infer GRNs from these datasets. Furthermore, we are interested in developing Transformer-based models to addressing various challenges in the analysis of scRNA-seq data analysis, including cell clustering, cell classification, and cell pseudo-timing analysis.

## Supplementary Material

btad165_Supplementary_Data
