# Supplementary material for: STGRNS: an interpretable transformer-based method for inferring gene regulatory networks from single-cell transcriptomic data
Source: Bioinformatics. 2023 Apr 2;39(4):btad165. doi: 10.1093/bioinformatics/btad165 (PMC10085635; doi:10.1093/bioinformatics/btad165)
Supplement: btad165_Supplementary_Data [file btad165_supplementary_data.zip › SupplementaryInformation.doc]

**SUPPLEMENTARY INFORMATION**

**STGRNS: An interpretable Transformer-based method for inferring gene regulatory networks from single-cell transcriptomic data**

**Jing Xu^1,2^, Aidi Zhang^1^, Fang Liu^1^, Xiujun Zhang^1,3*^**

### 1 Key Laboratory of Plant Germplasm Enhancement and Specialty Agriculture, Wuhan Botanical Garden, Chinese Academy of Sciences, Wuhan 430074, China

### 2 Center of Economic Botany, Core Botanical Gardens, Chinese Academy of Sciences, Wuhan 430074, China

### 3 University of Chinese Academy of Sciences, Beijing 100049, China

### * Correspondence: [zhangxj@wbgcas.cn](mailto:zhangxj@wbgcas.cn).

# SUPPLEMENTARY RESULTS

**Supplementary Figure 1. Comparison of CNNC_1_,CNNC_2_ and STGRNS.**

CNNC_1_: we used CNNC’s input generation method.

CNNC_2_: we used the input generation method modified from CNNC(Yuan and Bar-Joseph, 2019).

On the GRN reconstruction task, STGRNS performs better than CNNC_2_ on 97% (40/41) of the benchmarks in the term of AUROC and on 94% (38/41) of the benchmarks in the term of AUPRC. CNNC_2_ outperforms CNNC_1_ on all datasets. On the TF-gene causality prediction task, STGRNS wins on 71% (5/7)of datasets in the term of AUROC and on 86%(6/7) of datasets in the term of AUPRC; CNNC_2_ performs better than CNNC_1_ on 57%(4/7) of datasets in term of AUROC and on 71%(5/7) of datasets in term of AUPRC.On the TF-gene interaction task, STGRNS is the best model; CNNC_2_ performs better than CNNC_1_ on 57%(4/7) of datasets in terms of AUROC and AUPRC.On the GRN reconstruction task, the input generation method is very significant for the improvement of CNNC(***p < 0.001 by paired t-test. p = 3.55×10^-14^ in the term of AUROC and p = 1.07×10^-9^ in the term of AUPRC,n=41). However,the input generation method is not significant for the improvement of CNNC on the TF-gene prediction task (p = 0.80, 0.67 on the task of causality prediction; p = 0.45, 0.91 on the task of interaction prediction,n=7). In general, input generation is important for the performance of models.

(**a**) Comparison results on the top 500 highly variable genes datasets. (**b**) Comparison results on the top 1000 highly variable genes datasets. (**c**) Comparison results in the term of AUROC on the TF-gene prediction task. (**d**) Comparison results in the term of AUPRC on TF-gene prediction task.

**Supplementary Figure 2.** **Running time and consuming storage of generated data.**

In the first-class datasets, GEM consumes the least time and occupies the most storage on most datasets. In the second-class datasets, GEM consumes the most time and occupies the most storage on most datasets. For a gene pair (*Xi*, *Xj*), *Xi*, *Xj* are crisscross merged into *X_ij_* =( *X_i_*_,_*_j_*_, 0,_ *X_i_*_,_*_j_*_, 1,……,_ *X_i_*_,_*_j_*_,_*_k|s_*) as input to the model by GEM, so the smaller the number of cells, the less time and space it consumes. For each gene pair, scRNA-seq expression levels are transformed into 32 × 32 normalized empirical probability function (NEPDF) matrices in CNNC. We encode time-series scRNA-Seq data using a 2D or 3D histogram termed normalized empirical probability-density function (NEPDF) in TDL(Yuan and Bar-Joseph, 2021). The correlation of expression levels is measured by statistical criteria and pseudotimes information is preserved by sliding windows. For each gene pair, we extract a series of correlation vectors based on preprocessed single-cell transcriptomic data in DGRNS(Zhao, et al., 2022).

(**a**) Running time on the top 500 variable gene datasets. (**b**) Running time on the top 1000 highly variable gene datasets. (**c**) Running time on the second-class datasets. (**d**) The size of the top 500 highly variable gene datasets. (**e**) The size of the top 1000 highly variable gene datasets. (**f**)The size of the second-class datasets. (**g**) The time and storage consumed by the three methods for different cell numbers in the three datasets.

**Supplementary Figure 3. Training time**

In order to compare the training time of each method, all models are performed on one CPU(Intel(R) Xeon(R) CPU E5-2696 v3 @2.30GHz). In general, STGRNS runs at a moderate speed.

(**a**) Time spent on the top 500 highly variable genes datasets (**b**) Time spent on the top 1000 highly variable genes datasets (**c**) Time spent on the causality prediction task (**d**) Time spent on the interaction prediction task.

**Supplementary Figure 4. Comparison between methods without pseudo-time and STGRNS on various positive-negative ratio.**

At present, there are some methods developed for single-cell pseudo-time data, such as(Kuppe, et al., 2021; Sanchez-Castillo, et al., 2018). In order to study the influence of pseudo-time on the STGRNS and other methods, we discarded the pseudo-time information when generating the data. Results show that STGRNS performs better than the second-best methods on 95% (39/41) of the benchmarks in the term of AUROC and on 94% (38/41) of the benchmarks in the term of AUPRC, indicating that it can adapt to different types of datasets and achieve perfect performance. The pseudo-time information is not significant for STGRNS and CNNC (p > 0.05 by paired t-test. STGRNS: p=0.68 in term of AUROC, p=0.71 in term of AUPRC CNNC: p=0.77 in term of AUROC p=0.36 in term of AUPRC,n=41), however, it is significant for DGNRS (*p<0.05, p=0.02 in term of AUROC p=0.003 in term of AUPRC,n=41)

(**a**)Comparison results on the top 500 highly variable genes datasets (**b**) Comparison results on the top 1000 variable genes datasets.(**c**) Comparison results on the various positive-negative ratios. (**d**) Comparison results on the various down-sampling ratios.

**Supplementary Figure 5. The performance of STGRNS when the same TF presents in both training and test datasets.**

STGRNS_1_:The same TFs exists in the training and test datasets

STGRNS_2_:The same TFs does not exist in training dataset and test dataset.

If the same TFs exist in the training datasets and the test datasets, the performance of STGRNS will be significantly improved（***p < 0.005 by paired t-test. On the causality prediction task,p= 0.00093 in the term of AUROC p =0.00045 in the term of AUPRC; On the interaction prediction task,p=0.003616521 in the term of AUROC p =0.00292 in the term of AUPRC,n=7）

(**a**)The performance of STGRNS in the term of AUROC. (**b**) The performance of STGRNS in the term of AUPRC.

### **Supplementary Figure 6. The performance of each TF and the number of *a*>*b*,*a*<*b* in gene pairs (*a*,*b*) in the second-class datasets.**

### (**a**) hESC(2) (**b**) mESC(1) (**c**) mESC(2) (**d**) mESC (**e**) bone (**f**) dendritic

**Supplementary Figure 7. Transferability of STGRNS and CNNC on the task of TF-gene prediction task.**

We show the transfer ability of CNNC and STGRNS on the TF-gene prediction task. However, when training datasets and testing datasets are in different tasks, the transfer ability of CNNC is better than that of STGRNS, especially when the training task is in the causality prediction test task position interaction prediction.

**Supplementary Figure 8. Parameter sensitivity analysis.**

(**a**) The parameter for epoch ranges from 10 to 340 and each increment is 10. (**b**) This parameter of window size ranges from 10 to 490, and each increment is 10. (**c**) The parameter of head ranges from 1 to 200, and the parameter is divisible by input_dim. (**d**) The parameter of dropout ranges from 0.00 to 0.99, and the increment is 0.01. (**e**) The parameter of the batch size ranges from 10 to 4000. (**f**) The parameter of learning rate ranges from 0.0001 to 0.0199, and each increment is 0.0001. Performance is measured as AUROC and AUPRC.

**Supplementary Figure 9. Transferability of STGRNS on the task of GRNs reconstruction.**

Overall, the transferability of STGRNS on the task of GRN reconstruction is weak (**Supplementary table 4**) (**a**) Training in the 500_Non-specific-ChIP-seq-network_hHep and testing in other datasets. (**b**) Training in the 1000_Non-specific-ChIP-seq-network_hHep and testing in other datasets.

**Supplementary Figure 10. The perfermance of STGRNS_1,_ STGRNS_2 ,_STGRNS_3_,and Second.**

Second: The second-best method.

STGRNS_1_: having position encoding layer

STGRNS_2_: no position encoding layer

STGRNS_3_: gene pairs are first classified and then pooled

As Supplementary Figure 10a,b shown, position encoding and the position of the classification layer have little effect on the performance of STGRNS on the task of GRN reconstruction. The position of the classification layer has little effect on the performance of STGRNS, but position encoding has a great impact on the performance of STGRNS on the tasks of causality prediction and interaction prediction(**Supplementary Figure 10c,d**).

(**a**) Comparison results on the top 500 highly variable genes datasets. (**b**) Comparison results on the top 1000 highly variable genes datasets. (**c**) Comparison results on the TF-gene prediction task in the term of AUROC. (**d**) Comparison results on the TF-gene prediction task in the term of AUPRC.

**Pairwise model co**mparison results

| Task | metric | Comparation methods | result | P(paired t-test) |
| --- | --- | --- | --- | --- |
| GRN reconstruction | AUROC | STGRNS_1__ STGRNS_2_ | 39/41 | 3.75×10^-11^*** |
| GRN reconstruction | AUROC | STGRNS_1__ STGRNS_3_ | 38/41 | 5.86×10^-5^*** |
| GRN reconstruction | AUROC | STGRNS_1__Second | 39/41 | 3.56×10^-16^*** |
| GRN reconstruction | AUROC | STGRNS_2__Second | 38/41 | 6.83×10^-14^*** |
| GRN reconstruction | AUROC | STGRNS_3__Second | 38/41 | 4.22×10^-17^*** |
| GRN reconstruction | AUPRC | STGRNS_1__ STGRNS_2_ | 37/41 | 1.53×10^-7^*** |
| GRN reconstruction | AUPRC | STGRNS_1__ STGRNS_3_ | 27/41 | 0.019×10^-3^* |
| GRN reconstruction | AUPRC | STGRNS_1__Second | 40/41 | 4.92×10^-11^*** |
| GRN reconstruction | AUPRC | STGRNS_2__Second | 37/41 | 7.97×10^-10^*** |
| GRN reconstruction | AUPRC | STGRNS_3__Second | 38/41 | 2.67×10^-11^*** |
| causality prediction | AUROC | STGRNS_1__ STGRNS_2_ | 7/7 | 0.0038×10^-2^* |
| causality prediction | AUROC | STGRNS_1__ STGRNS_3_ | 5/7 | 0.21×10^-4^ |
| causality prediction | AUROC | STGRNS_1__Second | 7/7 | 0.044×10^-3^* |
| causality prediction | AUROC | STGRNS_2__Second | 1/7 | 0.062×10^-3^ |
| causality prediction | AUROC | STGRNS_3__Second | 4/7 | 0.45×10^-4^ |
| causality prediction | AUPRC | STGRNS_1__ STGRNS_2_ | 7/7 | 0.0025×10^-2^* |
| causality prediction | AUPRC | STGRNS_1__ STGRNS_3_ | 6/7 | 0.29×10^-4^ |
| causality prediction | AUPRC | STGRNS_1__Second | 7/7 | 0.081×10^-3^ |
| causality prediction | AUPRC | STGRNS_2__Second | 1/7 | 0.067×10^-3^ |
| causality prediction | AUPRC | STGRNS_3__Second | 4/7 | 0.49×10^-4^ |
| interaction prediction | AUROC | STGRNS_1__ STGRNS_2_ | 7/7 | 0.0085×10^-1^* |
| interaction prediction | AUROC | STGRNS_1__ STGRNS_3_ | 6/7 | 0.062×10^-3^ |
| interaction prediction | AUROC | STGRNS_1__Second | 7/7 | 0.021×10^-3^* |
| interaction prediction | AUROC | STGRNS_2__Second | 2/7 | 0.28×10^-4^ |
| interaction prediction | AUROC | STGRNS_3__Second | 6/7 | 0.042×10^-3^* |
| interaction prediction | AUPRC | STGRNS_1__ STGRNS_2_ | 7/7 | 0.0057* |
| interaction prediction | AUPRC | STGRNS_1__ STGRNS_3_ | 6/7 | 0.15×10^-4^ |
| interaction prediction | AUPRC | STGRNS_1__Second | 7/7 | 0.028×10^-3^* |
| interaction prediction | AUPRC | STGRNS_2__Second | 1/7 | 0.068×10^-3^ |
| interaction prediction | AUPRC | STGRNS_3__Second | 5/7 | 0.49×10^-4^ |

**Supplementary Figure 11. Performance of STGRNS with limited training cell.**

We selected mESC, bone, and dendritic as templates to validate the model's performance against varying cell numbers of different sizes. Overall, causality inference is more affected by cell number than interaction inference for all models. For causality inference, STGRNS is more affected by the number of cells than other models.

(**a-c**) The performance of STGRNS with the limited number of training cells compared to

other methods on the task of interaction prediction. (**d-f**) The performance of STGRNS with the limited number of training cells compared to other methods on the task of causality prediction.

**Supplementary Figure 12. Patch are your need.**

Second: The second-best method.

STGRNS_1_: having Encoder Layer

STGRNS_2_: no Encoder Layer

STGRNS_3_: no Encoder Layer and position encoding layer

The question that whether the perfect performance of ViT(Dosovitskiy, et al., 2020) is due to its powerful Transformer architecture, or due to the use of patch as input representation is asked on ConvMixer(Trockman and Kolter, 2022). ConvMixer outperformed classical vision models (such as ResNet(He, et al., 2016)) and performed better than ViT on some datasets. Inspired by this, we remove the transformer and use only two linear layers to verify the model's performance(**Supplementary Figure 12** **e middle**). Besides, we also removed the positional encoding without the encoder layer(**Supplementary Figure 12** **e left**).

Even without the transformer architecture, STGRNS still outperforms other models in the vast majority of datasets, showing that GEM plays a big role. In addition, the transformer architecture also significantly improves the performance of the model.

(**a**) Comparison results on the top 500 datasets. (**b**) Comparison results on the top 1000 datasets. (**c**) Comparison results on TF-gene prediction task in the term of AUROC. (**d**) Comparison results on TF-gene prediction task in the term of AUPRC.

Pairwise model comparison results

| Task | metric | Comparation methods | result | P(paired t-test) |
| --- | --- | --- | --- | --- |
| GRN reconstruction | AUROC | STGRNS_1__ STGRNS_2_ | 39/41 | 1.89×10^-10^*** |
| GRN reconstruction | AUROC | STGRNS_1__ STGRNS_3_ | 40/41 | 9.01×10^-12^*** |
| GRN reconstruction | AUROC | STGRNS_2__ STGRNS_3_ | 26/41 | 0.04×10^-4^* |
| GRN reconstruction | AUROC | STGRNS_1__Second | 39/41 | 3.56×10^-16^*** |
| GRN reconstruction | AUROC | STGRNS_2__Second | 38/41 | 1.39×10^-10^*** |
| GRN reconstruction | AUROC | STGRNS_3__Second | 37/41 | 1.39×10^-10^*** |
| GRN reconstruction | AUPRC | STGRNS_1__ STGRNS_2_ | 36/41 | 9.03×10^-8^*** |
| GRN reconstruction | AUPRC | STGRNS_1__ STGRNS_3_ | 34/41 | 2.39×10^-7^*** |
| GRN reconstruction | AUPRC | STGRNS_2__ STGRNS_3_ | 20/41 | 0.47×10^-4^ |
| GRN reconstruction | AUPRC | STGRNS_1__Second | 40/41 | 4.92×10^-11^*** |
| GRN reconstruction | AUPRC | STGRNS_2__Second | 36/41 | 5.06×10^-6^*** |
| GRN reconstruction | AUPRC | STGRNS_3__Second | 37/41 | 5.06×10^-6^*** |
| causality prediction | AUROC | STGRNS_1__ STGRNS_2_ | 6/7 | 0.05×10^-4^ |
| causality prediction | AUROC | STGRNS_1__ STGRNS_3_ | 5/7 | 0.049×10^-3^* |
| causality prediction | AUROC | STGRNS_2__ STGRNS_3_ | 3/7 | 0.35×10^-4^ |
| causality prediction | AUROC | STGRNS_1__Second | 7/7 | 0.04×10^-4^* |
| causality prediction | AUROC | STGRNS_2__Second | 3/7 | 0.38×10^-4^ |
| causality prediction | AUROC | STGRNS_3__Second | 2/7 | 0.18×10^-4^ |
| causality prediction | AUPRC | STGRNS_1__ STGRNS_2_ | 5/7 | 0.06×10^-4^ |
| causality prediction | AUPRC | STGRNS_1__ STGRNS_3_ | 5/7 | 0.029×10^-3^* |
| causality prediction | AUPRC | STGRNS_2__ STGRNS_3_ | 4/7 | 0.31×10^-4^ |
| causality prediction | AUPRC | STGRNS_1__Second | 7/7 | 0.08×10^-4^ |
| causality prediction | AUPRC | STGRNS_2__Second | 3/7 | 0.37×10^-3^ |
| causality prediction | AUPRC | STGRNS_3__Second | 3/7 | 0.16×10^-4^ |
| interaction prediction | AUROC | STGRNS_1__ STGRNS_2_ | 7/7 | 0.00047×10^-1^*** |
| interaction prediction | AUROC | STGRNS_1__ STGRNS_3_ | 7/7 | 0.00076×10^-1^*** |
| interaction prediction | AUROC | STGRNS_2__ STGRNS_3_ | 3/7 | 0.43×10^-4^ |
| interaction prediction | AUROC | STGRNS_1__Second | 7/7 | 0.02×10^-4^* |
| interaction prediction | AUROC | STGRNS_2__Second | 2/7 | 0.88×10^-4^ |
| interaction prediction | AUROC | STGRNS_3__Second | 3/7 | 0.64×10^-4^ |
| interaction prediction | AUPRC | STGRNS_1__ STGRNS_2_ | 5/7 | 0.034×10^-3^* |
| interaction prediction | AUPRC | STGRNS_1__ STGRNS_3_ | 7/7 | 0.026×10^-3^* |
| interaction prediction | AUPRC | STGRNS_2__ STGRNS_3_ | 3/7 | 0.73×10^-4^ |
| interaction prediction | AUPRC | STGRNS_1__Second | 7/7 | 0.028×10^-3^* |
| interaction prediction | AUPRC | STGRNS_2__Second | 3/7 | 0.75×10^-3^ |
| interaction prediction | AUPRC | STGRNS_3__Second | 2/7 | 0.89×10^-4^ |

# Data Availability

For the GRN reconstruction task, the processed experimental single-cell gene expression datasets are available on Zenodo at [https://doi.org/10.5281/zenodo.3378975](https://doi.org/10.5281/zenodo.3378975/). The time-series scRNA-Seq data from four mouse and human embryonic stem cell studies (mESC and hESC) used for the TF-gene prediction task is available with the accession numbers of GSE79578, GSE65525, E-MTAB-3929, and GSE75748(Chu, et al., 2016; Klein, et al., 2015; Petropoulos, et al., 2016; Semrau, et al., 2017). The gene expression and ChIP-Seq data of bone marrow-derived macrophages, dendritic cells, and mESC(1) are available at <https://github.com/xiaoyeye/CNNC>.

# Supplementary Note

**Supplementary Note 1: Gene expression motif**

Motif refers to a local conserved region in a sequence, or a small sequence pattern shared by a group of sequences. DNA sequence motifs can be defined as transcription factor binding sites. we get the binding sites of a TF by known target genes of this TF. When we judge whether a gene is the target gene of this TF, we search the promoter region upstream of the gene. If the sequence of the binding characteristics can be searched, the TF may regulate this gene. In addition, genes with similar expression profiles are simultaneously activated or repressed in specific tissues and developmental stages and these genes may be regulated by common transcription factors.

Inspired by the two theories, we propose a gene expression motif. We divide the gene pair into sub-vectors as the input of the model, and the purpose is to learn the motif of gene expression vectors from the known gene pairs with the same transcription factor through the model. The model focus on the motif in the predicted gene pair to determine whether the unknown gene pairs are interaction. We also proved this by interpreting the results predicted by STGRNS, and we also proved that when the same transcription factor exists in the training datasets and test datasets, the predictive ability of the model will be greatly improved.

**Supplementary Note 2: The Input generation method in CNNC**

For each gene pair, scRNA-seq expression levels are transformed into 32×32 normalized empirical probability function (NEPDF) matrices in CNNC.

**Supplementary Note 3: The Input generation method in DGRNS**

The correlation of expression levels is measured by statistical criteria and pseudotimes information is preserved by sliding windows. For each gene pair, we extract a series of correlation vectors based on preprocessed single-cell transcriptomic data in DGRNS.

**Supplementary Note 4: The architecture of CNNC**

**Supplementary Note 5: The architecture of DGRNS**

**Supplementary Note 6: TDL**

**The structure of 3D CNN network.**

**The structure of stacked conv LSTM network.**

**References**

Chu, L.F.*, et al.* Single-cell RNA-seq reveals novel regulators of human embryonic stem cell differentiation to definitive endoderm. *Genome Biol* 2016;17(1):173.

Dosovitskiy, A.*, et al.* An image is worth 16x16 words: Transformers for image recognition at scale. *arXiv* 2020:11929.

He, K.*, et al.* Deep residual learning for image recognition. In, *Proceedings of the IEEE Conference on Computer Vision and Pattern Recognition (CVPR)*. 2016. p. 770-778.

Klein, A.M.*, et al.* Droplet barcoding for single-cell transcriptomics applied to embryonic stem cells. *Cell* 2015;161(5):1187-1201.

Kuppe, C.*, et al.* Decoding myofibroblast origins in human kidney fibrosis. *Nature* 2021;589(7841):281-286.

Petropoulos, S.*, et al.* Single-Cell RNA-Seq Reveals Lineage and X Chromosome Dynamics in Human Preimplantation Embryos. *Cell* 2016;165(4):1012-1026.

Sanchez-Castillo, M.*, et al.* A Bayesian framework for the inference of gene regulatory networks from time and pseudo-time series data. *Bioinformatics* 2018;34(6):964-970.

Semrau, S.*, et al.* Dynamics of lineage commitment revealed by single-cell transcriptomics of differentiating embryonic stem cells. *Nat Commun* 2017;8(1):1096.

Trockman, A. and Kolter, J.Z. Patches Are All You Need? *arXiv* 2022:2201.09792.

Yuan, Y. and Bar-Joseph, Z. Deep learning for inferring gene relationships from single-cell expression data. *Proc Natl Acad Sci U S A* 2019;116(52):27151-27158.

Yuan, Y. and Bar-Joseph, Z. Deep learning of gene relationships from single cell time-course expression data. *Brief Bioinform* 2021;22(5):bbab142.

Zhao, M.*, et al.* A hybrid deep learning framework for gene regulatory network inference from single-cell transcriptomic data. *Brief Bioinform* 2022;23(2):bbab568.
